# The genetics of breast cancer risk in the post-genome era: thoughts on study design to move past *BRCA* and towards clinical relevance

**DOI:** 10.1186/s13058-016-0759-4

**Published:** 2016-10-03

**Authors:** Andrew D. Skol, Mark M. Sasaki, Kenan Onel

**Affiliations:** 1 Department of Pediatrics, The University of Chicago, Chicago, IL 60637 USA; 2 Department of Biology, Hamilton College, Clinton, NY 13323 USA; 3 Section of Hematology/Oncology, Department of Pediatrics, The University of Chicago, KCBD 5140, 900 East 57th Street, Chicago, IL 60637 USA

**Keywords:** Genetics, Genome-wide association study, Genomics, Linkage analysis, Next-generation sequencing, Penetrance, Predisposition

## Abstract

More than 12 % of women will be diagnosed with breast cancer in their lifetime. Although there have been tremendous advances in elucidating genetic risk factors underlying both familial and sporadic breast cancer, much of the genetic contribution to breast cancer etiology remains unknown. The discovery of *BRCA1* and *BRCA2* over 20 years ago remains the seminal event in the field and has paved the way for the discovery of other high-penetrance susceptibility genes by linkage analysis. The advent of genome-wide association studies made possible the next wave of discoveries, in which over 80 low-penetrance and moderate-penetrance variants were identified. Although these studies were highly successful at discovering variants associated with both familial and sporadic breast cancer, the variants identified to date explain only 50 % of the heritability of breast cancer. In this review, we look back at the investigative strategies that have led to our current understanding of breast cancer genetics, consider the challenges of performing association studies in heterogeneous complex diseases such as breast cancer, and look ahead toward the types of study designs that may lead to the identification of the genetic variation accounting for the remaining missing heritability.

## Background

Among women, breast cancer accounts for over 25 % of cancer diagnoses and 15 % of cancer-related deaths [1]. Ten percent of women with breast cancer have a family history of the disease [2]. Compared with women without a family history, women with one premenopausal first-degree relative with breast cancer are at 3.3-fold greater risk, and women with two first-degree relatives with breast cancer are at 3.6-fold greater risk [3], demonstrating that germline genetics contributes significantly to risk.

To identify genetic factors associated with breast cancer predisposition, early studies used linkage analysis and positional cloning in families with multiple affected individuals to discover highly penetrant susceptibility genes such as *BRCA1* and *BRCA2* [4, 5]. Although these studies were successful and could explain about 20 % of the familial aggregation of breast cancer risk [6], they provided little insight into the role of genetics in nonfamilial breast cancer.

More recently, genome-wide association studies (GWAS) have identified over 80 loci significantly associated with sporadic breast cancer. Collectively, however, these variants explain only 16 % of breast cancer heritability [7]. The inability of GWAS to identify a greater proportion of the genetic risk stems from many factors, including genotyping platform limitations in interrogating rare variation. Consequently, attention has shifted recently to investigating the impact of rare variation on disease, motivated in part by the precipitous drop in next-generation sequencing (NGS) costs.

Here, we will review linkage studies, GWAS, and NGS studies that have led to our current knowledge of the genetics of breast cancer susceptibility. Our emphasis will be on the strengths and limitations of different study designs with the potential to yield clinically translatable discoveries.

### Family linkage studies and rare high-penetrance and medium-penetrance risk variants

Clinically, the most important breast cancer susceptibility genes are *BRCA1* and *BRCA2*. The loci where these genes reside were first observed as linkage peaks on chromosomes 17q21 and 13q12, in studies of just 23 and 15 families [8, 9]. The genes were identified shortly thereafter by fine-mapping using linkage analysis of more families [10], followed by positional cloning [4, 11] and mutation screening [5]. All told, 5 % of all breast cancer cases and up to 25 % of familial breast cancer cases can be attributed to high-penetrance mutations in *BRCA1* and *BRCA2* [12].

The impact of mutations in either gene can be dramatic; 65 % and 45 % of women with deleterious mutations in *BRCA1* or *BRCA2*, respectively, will develop breast cancer by age 70 [13], and the risk increases to 85 % and 84 %, respectively, for women with a family history of breast cancer [14, 15]. Generally, *BRCA* susceptibility variants identified in breast cancer patients with a positive family history are unique to each family. However, founder mutations are observed within certain populations. For example, in Ashkenazi Jews, the *BRCA2* c.5946delT (previously 6174delT) mutation is found at an allele frequency (AF) of 0.009–0.015 [16].

There are other genes in which germline mutations have been identified that substantially increase the risk of breast cancer. Most of these were initially discovered because they cause a syndrome of which breast cancer is a component. Li–Fraumeni syndrome (LFS), for example, is a cancer predisposition syndrome due to germline mutations in *TP53*, in which the most common cancer type in women is breast cancer. Malkin et al. [17] initially investigated a link between *TP53* and LFS because somatic mutations in *TP53* were identified in cancer types commonly observed in LFS families. They sequenced *TP53* exons 5–8 in five LFS families because this region contains the highly conserved DNA binding domain and harbors most *TP53* somatic mutations. Affected members of all families were found to have segregating germline mutations in this region, with inheritance consistent with a dominant model.

A deletion in *CHEK2* was investigated by the same group in 1071 breast cancer patients from 718 families with a positive history of breast cancer and no *BRCA* mutation, a population-based set of 636 patients, and 1620 controls. They found the CHEK2*1100delC variant at a frequency of 1.1 % in controls, 5.1 % in cases with a family history, and 13.5 % in cases with a family history of male breast cancer [18]. Intriguingly, the AF in nonfamilial breast cancer cases did not differ from that of controls (1.4 %).

A similar strategy of examining genes causing syndromes with a high incidence of breast cancer led to the discovery of *PALB2*. Biallelic *PALB2* mutations cause a Fanconi anemia (FA) phenotype similar to that caused by biallelic *BRCA2* mutations. Rahman et al. [19] investigated whether heterozygous *PALB2* mutation carriers, like *BRCA2* carriers, were at increased breast cancer risk. They sequenced *PALB2* in 1084 controls and 923 cases with a family history of breast cancer but no *BRCA* mutation. They found 10 *PALB2* truncating mutations among cases, but none among controls. More recently, Antoniou et al. [20] examined the breast cancer risk in 362 members of 154 families with loss of function mutations in *PALB2*. They found an age-dependent trend in breast cancer risk among *PALB2* mutation carriers relative to age-matched controls (age 40–44 years, RR = 8.02; age 50–54, RR = 6.55; age 60–64, RR = 5.45). Interestingly, women with *PALB2* mutations from families with a history of breast cancer had substantially greater breast cancer risk than women with *PALB2* mutations but no family history.

*BRIP1* also causes FA when deleted biallelically. *BRIP1* was investigated as a breast cancer susceptibility gene in heterozygous carriers because it interacts with other breast cancer predisposing genes such as *BRCA1*. Seal et al. [21] sequenced the exons and exon–intron boundaries of *BRIP1* in 1212 breast cancer cases with a family history of disease and no *BRCA* mutation and in 2081 controls, and found mutations in nine cases (0.74 %) but only in two controls (0.10 %). Intriguingly, no *BRIP1*-mutated FA family had a family history of breast cancer. More recently, Easton et al. [22] sequenced the coding region of *BRIP1* in more than 13,000 population-based breast cancer cases and 8000 controls, and found no excess of truncating mutations in cases relative to controls (0.21 % vs 0.23 %, respectively). The apparently discrepant results between these two studies may be another example of the importance of family history in determining the penetrance of a risk variant. However, these results also illustrate the challenges inherent in drawing conclusions about rare variants of modest effect, even when analyzing tens of thousands of samples.

*ATM*, in which biallelic mutations cause ataxia-telangiectasia (AT), was also suspected to be a breast cancer susceptibility gene in carriers because of an increased breast cancer incidence among relatives of AT patients. Renwick et al. [23] sequenced *ATM* in 443 *BRCA*-negative cases from families with at least three breast cancer-affected members and in 521 controls. Nine truncating and exon-skipping mutations were identified in cases, while only two were found in controls. All mutations found in cases were predicted to cause AT, and seven had been observed previously in AT cases. Another group performed a meta-analysis using *ATM* sequence data from 1544 breast cancer cases and 1224 controls [24]. They found only marginal evidence for an excess of truncating and splice site variants within cases relative to controls, but greater evidence when restricting attention to variants with the greatest evidence of evolutionary constraint. Bernstein et al. [25] performed an *ATM* mutation screen in 708 unilateral breast cancer survivors who developed contralateral breast cancer following radiotherapy and 1397 who did not. They found that women with AT-associated *ATM* mutations treated previously with radiation had significantly greater risk of contralateral breast cancer than unexposed women either with no mutation (Gy < 1.0, RR = 2.8; Gy ≥ 1.0, RR = 3.3) or unexposed women with the same mutation (Gy < 1.0, RR = 5.3; Gy ≥ 1.0, RR = 5.8). These studies suggest that *ATM* mutations causing AT but not other *ATM* variants are associated with increased breast cancer risk in heterozygous carriers and that this risk may be increased by radiation exposure; however, these results await replication, and current guidelines do not recommend that heterozygous *ATM* mutation carriers should avoid radiation.

Some genes are uniquely associated with risk for specific breast cancer subtypes. *CDH1*, for example, is a tumor suppressor mutated in invasive lobular carcinoma of the breast (ILCB) but not ductal breast cancer [26]. Because germline mutations in *CDH1* cause hereditary diffuse gastric cancer (HDGC) and HDGC patients have a high incidence of ILCB (50 % lifetime risk) [27], Pharoah et al. [28] investigated the penetrance of *CDH1* germline mutations by performing segregation analysis in 11 families with at least three HDGC cases and a confirmed *CDH1* mutation. They estimated the cumulative risk of HDGC and ILCB by age 80 among women in these families to be 83 % and 39 %, respectively.

In summary, high-penetrance and moderate-penetrance variants in these genes collectively explain approximately 20 % of the familial risk of breast cancer [29]. Undoubtedly, continued investigation of families with multiple cancer-affected members will lead to the identification of other variants in these genes that also predispose to breast cancer, and will also shed light on the penetrance of these variants. Additionally, as the true prevalence of other cancer-predisposing syndromes becomes clear, it is likely that new associations between these syndromes and increased breast cancer risk will be discovered. Importantly, two themes are emerging from family studies that have important clinical and research implications. First, there is growing recognition that some variants causing heritable cancer syndromes when mutated biallelically also increase cancer risk among heterozygous carriers. Second, it is becoming increasingly clear that the contribution of some variants to breast cancer risk can be significantly modified by family history. Thus, there are clearly many lessons remaining to be learned through the continued study of familial breast cancer.

### Genome-wide association studies and common low-penetrance risk variants

Although rare high-penetrance mutations explain much of the genetic breast cancer risk in a small number of cases, they do not shed light on the role of genetics in nonfamilial breast cancer. There is, however, considerable evidence for a strong genetic contribution to risk even for sporadic breast cancer [30]. Most investigators believe that the genetic architecture of sporadic disease is polygenic, in which susceptibility results from the aggregate effect of many low-penetrance variants. GWAS are used to search for these variants by testing for AF differences in single nucleotide polymorphisms (SNPs) genotyped across the genome in a large sample of cases and healthy controls.

The first three breast cancer GWAS were published concurrently in 2007. In one of these studies, Stacey et al. [31] used 4554 cases and 17,577 controls of predominantly European ancestry (EA) to identify two common SNPs, rs13387042 and rs3803662, with odds ratios (ORs) of 1.2 and 1.28, respectively. In the second of these GWAS, Easton et al. [32] identified five independent susceptibility loci in EA individuals using 4398 breast cancer cases and 4316 controls in a discovery stage, and more than 20,000 cases and 20,000 controls in a confirmation stage. These loci contained SNPs in or near *FGFR2*, *TNRC9*, *MAP3K1*, *LSP1*, and *H19*. Finally, Hunter et al. [33] conducted a two-stage genome-wide association study using 2921 European postmenopausal breast cancer cases and 3214 controls, and identified four intronic *FGFR2* SNPs, thereby independently replicating Easton et al.’s finding.

To date, more than 60 breast cancer GWAS have been performed [34]. As this number grows, the advantage of meta-analysis—the combining of evidence across multiple studies—becomes obvious. The first large-scale meta-analysis, conducted by Michailidou et al. [35] in 2013, employed 55,342 EA cases and 54,455 controls from nine GWAS and identified 41 new susceptibility loci. Two years later, an even larger meta-analysis, comprising more than 120,000 individuals from 52 studies, found 15 more susceptibility loci [7], bringing the current number of susceptibility loci identified by GWAS to 84.

Many variants in GWAS show consistent associations across populations; apparent population-specific associations can often be explained by differences in AF among populations. For example, in 2016 African American (AA) breast cancer patients and 2745 controls, 36 of 47 (67 %) EA breast cancer risk SNPs had ORs in AA in the same direction, and seven (15 %) had nominally significant *P* values [36]. In East Asian women (23,637 cases and 25,579 controls), 31 of 67 EA susceptibility loci were significantly associated with breast cancer. Thus, variants contributing to sporadic breast cancer risk are likely to be similar across ancestries.

Typically, a homogeneous disease model is assumed in genetic studies, and cases are lumped together because of the increase in power that comes with increased sample size. Splitting cases by subtype is an alternative study design, with the potential to increase power despite decreasing sample size.

There have been GWAS investigating specific breast cancer subtypes based on the presence or absence of estrogen receptor (ER), progesterone receptor (PR), and/or HER2 expression [37]. Broeks et al. [38] and Figueroa et al. [39] investigated 10 validated SNPs for heterogeneity of effect size between ER+ and ER– patients. They found that seven SNPs had significantly larger effects in ER+ patients than in ER– patients, and only two SNPs remained associated with ER– breast cancer after adjusting for multiple testing. Stevens et al. [37] studied 65 validated breast cancer variants and found that while 38 were associated with both ER+ and ER– disease, the rest were unique to only one subtype. Recently, three meta-analyses of ER– breast cancer were performed that identified seven risk loci specific to this disease subtype [38, 40, 41]. Although no subtype-specific association had a particularly large effect size, these results suggest that subsetting cases based upon clinical or molecular characteristics may be an important strategy for future investigations.

While the 80+ breast cancer-associated loci identified to date have greatly expanded our knowledge of the genetics of the disease, they also have the potential to be of clinical utility. Recent studies have assessed the clinical utility of variants in GWAS using the polygenic risk score (PRS), a crude estimate of a patient’s OR for disease calculated by summing the ORs for each risk allele carried by the patient [42–45]. In one study, Mavaddat et al. [42] used the PRS in a logistic regression model to demonstrate that the OR for disease differed significantly between patients with a PRS in either the highest or lowest one percentile as compared with patients with an average PRS (OR_1%_ = 0.32, OR_99%_ = 3.36). The discriminative accuracy of the PRS as measured by a *C*-statistic, however, was modest (*C* = 0.62). The authors estimated that the lifetime risk of cancer for women below the first and above the 99th percentile of the PRS is 3.5 % and 29.0 %, respectively. In the UK, enhanced surveillance is recommended for women with both a family history of breast cancer and a lifetime risk of breast cancer above 17 %. Using the PRS, about 8 % of UK women at this risk level—accounting for about 17 % of breast cancer cases—can be identified. Thus, risk assessment can be marginally improved by incorporating susceptibility variants from GWAS. Although variants in GWAS currently have little impact on public health, this is likely to change in the future.

### Next-generation sequencing and rare variation

Taken together, these results suggest that lumping and splitting strategies for GWAS are unlikely to identify much of the missing non-high- or non-moderate-penetrance genetic contribution to breast cancer risk. One explanation for this is that GWAS are designed to identify common variants (MAF > 0.01), and to only poorly interrogate rare variants (MAF < 0.01) [46]. Thus, much of the rare variation in the genome remains uninvestigated. That rare variants may contribute significantly to risk is an appealing hypothesis because variants strongly predisposing to disease should be associated with lower fitness and be maintained at low AFs due to purifying selection. NGS can directly interrogate every position in the genome, and therefore identifies both common and rare variation. Consequently, many investigators have turned to NGS to study rare variants in complex diseases.

NGS approaches can be divided into four broad experimental strategies: sequence large numbers of unrelated patients and healthy controls to identify rare variants with AFs differing significantly between cases and controls; perform a staged study in which unrelated individuals from high-risk families meeting certain criteria (e.g., no identified mutations in *BRCA1* or *BRCA2*) are sequenced in stage one, and identified candidate risk variants are genotyped in a much larger set of cases and controls in stage two; perform a staged study in which unrelated individuals sharing critical clinical or other characteristics, such as driver somatic mutations, are sequenced in stage one, and candidate variants are subsequently genotyped in stage two; and sequence multiple related affected individuals from families enriched for disease to identify novel candidate variants and/or genes, and then interrogate these variants and genes in large case–control sets.

In the first strategy, when comparing case–control AF differences, the comparisons can be at a specific chromosomal position, in aggregate within a single gene, or in aggregate across multiple genes within a molecular or functional pathway [47]. These studies are followed up in large numbers of cases and controls investigating only genes with evidence for association.

This study design is essentially that of GWAS, except that the number of variants tested is reduced and the AF spectrum is shifted from common to rare. There are, however, statistical issues with this approach that significantly reduce its power. First, even when restricting attention to rare variation, tens of thousands of variants are tested for association. Second, the AF of the variants tested profoundly influences their power to be detected. Consider three SNPs with the same effect size but AFs of 0.10, 0.01, and 0.001. If a study using 700 cases and 700 controls has 80 % power to identify a risk allele with AF = 0.10, then 5910 cases and 5910 controls are required for the same power to identify the risk allele with AF = 0.01, and 58,130 cases and 58,130 controls are required for the risk allele with AF = 0.001.

As an example of a study utilizing this design, Flannick et al. [48] interrogated exonic variants in 115 type 2 diabetes genes by sequencing 758 Scandinavian cases and controls selected from phenotypic extremes, and found no evidence for association. They then genotyped 71 rare variants that either had nominal significance or were predicted to affect protein structure in 13,884 individuals, and still found no evidence for association. They subsequently followed up a single variant in *SLC30A8* in an additional 33,000 individuals, and found it was nominally significant. These results are quite sobering, given the enormous sample size needed to discover only a single rare variant, as well as the ad-hoc criteria employed for variant selection.

To overcome these barriers, the second NGS study design is a staged study in which unrelated cases selected for a presumed high “genetic load” for disease are sequenced in stage one, and only variants with evidence for association are genotyped in stage two in a much larger number of cases and controls. This approach assumes that the genetic complexity in patients with high “genetic load” is considerably reduced as compared with unselected patients because of the high-penetrance mutations. One study using this approach was performed by Cybulski et al. [49], who sequenced the exomes of women with familial breast cancer from two populations harboring founder mutations, Quebec-based French-Canadians and Poles. A total of 195 patients were selected based on family history or early age of breast cancer diagnosis and no mutation in *BRCA*, *CHEK2*, *NBN*, or *PALB2*. Multiple rare truncating variants were found in *RECQL*, a previously identified cancer-related gene, in both populations. Fourteen *RECQL* exons were then sequenced in 950 *BRCA*-negative Polish and French-Canadian familial breast cancer patients. Two previously unknown germline truncating mutations were discovered in four patients; one only in Polish individuals, and the other only in French-Canadian individuals. The Polish mutation was then genotyped in 13,136 unselected Polish cases and 4702 controls, and the French-Canadian mutation in 538 French-Canadian cases with familial or early-onset breast cancer and 7136 controls. In the Polish set the risk AF was 0.23 % in cases and 0.04 % in controls (*P* = 0.008), while in the French-Canadian set the frequencies were 0.69 % and 0.014 %, respectively (*P* = 3 × 10^–6^). Thus, by performing a discovery stage using cases who, based on clinical characteristics and/or family history, were likely to have high-penetrance mutations, the number of hypotheses tested in a subsequent validation stage was limited, thereby minimizing the penalty for multiple testing.

The purpose of selecting cases based on their presumed genetic load is to reduce the genetic complexity of the analysis. This concept can be expanded to other clinical or genetic features, which forms the basis of the third NGS study design. For example, in cancer the presence of a tumor genome provides the opportunity to use the mutational landscape of a patient’s somatic genome as supportive evidence to guide discovery of novel candidate germline risk variants, as Kanchi et al. [50] demonstrated in ovarian cancer.

Other studies have also shown relationships between the tumor genome and specific germline predispositions. Liu et al. [51], for example, demonstrated in patients with nonsmall-cell lung cancer that known functional germline polymorphisms in *EGFR* predict both higher somatic mutational burden and also specific somatic exonic microdeletions within *EGFR*. Additionally, Rausch et al. [52] found that chromothripsis (the occurrence of massive somatic chromosomal rearrangements within localized regions of the genome) in some, but not all, cancers, was associated with the presence of high-penetrance germline mutations in *TP53* that have been associated with LFS. Similarly, in breast cancer the recognition that some subtypes are enriched for germline deleterious mutations in predisposition genes can help prioritize individuals and families for NGS investigations either to identify known or to discover novel high-penetrance risk variants even in the absence of family history [53].

Finally, building upon the idea of leveraging genetically loaded individuals to improve power, the fourth NGS strategy is to sequence multigenerational families enriched for disease. Sequencing multiple affected family members as opposed to only probands simplifies considerably the analysis by limiting the number of candidate disease-causing mutations to those shared by affected family members and obligate carriers. Spurrell performed whole exome sequencing on 144 affected individuals from 54 breast cancer families with no germline mutations in known breast cancer genes to identify genes with truncating mutations shared by at least two affected family members. The study found germline mutations in *ATR*, *CHEK1*, and *GEN1* in three separate families. Another 2544 sporadic cases and 7652 controls were sequenced to identify additional rare variants in these three genes. An excess of truncating mutations in all three genes was found in cases, although the total number of cases with deleterious mutations was only 11 [54] (dissertation; not peer reviewed). A similar design was used by Kiiski et al. [55], who performed whole exome sequencing on 11 Finnish families enriched for breast cancer and identified 22 rare deleterious variants in 21 DNA repair genes. Of these, one variant in *FANCM* was significantly more common in a set of more than 3500 breast and ovarian cancer cases as compared with 2000 controls.

These examples illustrate the value of family context for genetic studies. If a gene harbors a mutation segregating in near-Mendelian fashion in one family, then there may be other highly penetrant mutations in the same gene in other families. Furthermore, if mutations identified in a family are also found in the general population, then an extension of this family-based study design is to investigate their contribution to sporadic disease risk. As noted for both the linkage and candidate gene examples discussed earlier, family history can modify the contribution to risk of even highly penetrant variants. This may suggest the existence of genetic modifiers in families that potentiate the effect of risk alleles in these families, or attenuate their affect in unaffected mutation carriers. Thus, these studies may provide the best opportunity to convert insights from rare variants into discoveries of clinical and biological significance.

An important caveat to NGS studies is that they are not agnostic; all approaches assume that variants must be filtered based on functional or population characteristics. This limits analysis only to variants with high a-priori likelihood of being functionally important, reducing the number investigated and the burden of multiple testing. Without filtering, all NGS studies would be woefully underpowered. Filters include: minor AF; functional consequence (nonsynonymous, missense, nonsense, splice-site, frame-shift indel); and functional annotation, such as predicted importance for protein function (SIFT [56], Polyphen-2 [57]), conservation across species (GERP [58], PhyloP [58, 59], and SiPhy [60]), or overall predicted importance abstracted from multiple sources (MutationTaster [61], CADD [62]). In some studies, further filtering restricts attention to genes or pathways previously implicated in disease.

Another important consideration is that not all familial disease aggregates are due to high-penetrance mutations. This is especially true for common diseases such as breast cancer, in which it is not unusual to observe familial clusters simply by chance. Additionally, nongenetic factors such as shared environment also contribute to familial risk, independent of genetics.

### Risk variants in context: the role of environment

Breast cancer, like all complex diseases, results from genetic factors, environmental factors, and interactions between the two. While it is clear that the environment contributes significantly to risk, it is unclear how to incorporate environment into genetic models. Because exposures are essentially impossible to quantify for any individual, genetic association studies largely disregard them. The inevitable consequence is that cases arising via multiple distinct mechanisms are lumped and analyzed together, resulting in an overall attenuation of genetic signals. In rare instances when an etiologic exposure is known and measured, restricting case and control selection to those with the exposure is an effective strategy both to account for this exposure as a risk factor and to simplify the underlying genetic vulnerability. While this study design yields smaller studies, the fact that cases and controls are more homogeneous improves power to detect exposure-specific risk variants [63].

In practice, matching exposures is challenging and can be confounded by changes over time in the contribution of an exposure to disease risk. Nonetheless, there are opportunities to design studies controlling for exposure. Adolescents with Hodgkin lymphoma (HL) treated with ionizing radiation (IR) are at high risk for IR-induced second primary tumors, particularly breast cancer [64, 65]. Best et al. [66] hypothesized that because both cases (HL survivors who did develop a second cancer) and controls (HL survivors who did not develop a second cancer) were exposed to IR, the major factors distinguishing cases from controls were genetic. They performed discovery GWAS using just 96 cases and 82 controls, and succeeded in identifying and replicating a variant that they then demonstrated functionally regulates IR-mediated *PRDM1* induction. Importantly, although this variant was highly penetrant in the context of IR, it did not contribute to risk in the absence of IR. These results underscore the importance of environmental context in genetic studies. Success in designing studies incorporating exposures, however, is predicated upon a substantive epidemiological understanding of the role of these environmental factors in disease susceptibility.

## Conclusion

The search for germline breast cancer predisposing variants has been highly successful. Linkage analysis and fine-mapping led to the discovery of *BRCA1/2* and other high-penetrance and medium-penetrance genes, which are mutated in about a quarter of all familial breast cancer cases. Eighty-four independent loci have been identified by GWAS, which account for a small but meaningful proportion of sporadic breast cancer risk. New technologies such as NGS have catalyzed a new era of discovery by making possible studies unimaginable even a few years ago.

With new technologies, however, come new challenges, perhaps foremost of which is the urgent need to rethink study design. Complex diseases such as breast cancer lie at the interface of human genetics and epidemiology. Here, we propose that one powerful framework for future investigation may be studying the genetics of breast cancer risk in context; in particular, the context of either a homogeneous exposure or familiality. An important implication of epidemiology-guided study design is that the contribution of variants to disease is not monolithic; variants that are highly penetrant in one context may not be associated with disease in another.

Finally, genetics is predicated upon collaborative research. The sine qua non is shared access to large numbers of samples with well-annotated clinical and exposure data. However, genetics is only the starting point. Moreover, breakthroughs in breast cancer prevention and treatment can only come through functional follow-up of genetic discoveries. This is an exciting time for genetics, but only through concerted multidisciplinary efforts will the clinical promise of genetics will be realized.

## Abbreviations

AA: African American
AF: Allele frequency
AT: Ataxia-telangiectasia
EA: European American
ER: Estrogen receptor
FA: Fanconi anemia
GWAS: Genome-wide association studies
HDGC: Hereditary diffuse gastric cancer
HL: Hodgkin lymphoma
ILCB: Invasive lobular carcinoma of the breast
IR: Ionizing radiation
LFS: Li–Fraumeni syndrome
MAF: Minor allele frequency
NGS: Next-generation sequencing
OR: odds ratio
PR: Progesterone receptor
PRS: Polygenic risk score
SNP: Single nucleotide polymorphism
UK: United Kingdom
